# Premature Babies Can Be Cared for in the Maternity Ward without an Increased Risk and Discharged with a Feeding Tube If Necessary

**DOI:** 10.3390/children11040456

**Published:** 2024-04-10

**Authors:** Lea Rösch, Edda Hofstätter, Franziska Krasnitzer-Leitner, Martin Wald

**Affiliations:** Division of Neonatology, Department of Pediatrics and Adolescent Medicine, Paracelsus Medical University, Salzburg 5020, Austria

**Keywords:** late preterm infant, discharge, feeding tube, feeding difficulties

## Abstract

In general, premature babies are discharged home when they reach full self-feeding. We established a discharge management protocol which allows for discharging late preterm babies with a feeding tube if necessary. This retrospective study included 108 preterm infants (34+ weeks) born in 2019 and 2020. The preterm infants discharged with a feeding tube (*n* = 32) were born at 35.23 weeks’ gestation (±0.884), with a birth weight of 2423 g (±375.1), and were discharged at 7.22 days (±3.63) and had a weight of 3466 g (±591.3) at the first outpatient visit around the expected birth date. The preterm infants discharged without a feeding tube were born at 35.97 weeks’ gestation (±0.702) with a birth weight of 2589 g (±424.84), discharged home at 6.82 days (±7.11) and a weight of 3784 g (±621.8) at the first outpatient visit. The gestational week and birth weight were statistically significantly different between the groups, with a *p*-value of <0.001 for each, and the length of hospital stay (*p* = 0.762) and weight at follow-up (*p* = 0.064) did not significantly differ. No infant required tube-feeding at the time of the first outpatient visit, i.e., the time of expected birth. Therefore, with well-thought-out management, it is possible and safe to discharge preterm infants home with a feeding tube.

## 1. Introduction

At many neonatal units, achieving independent feeding is considered a necessary step before premature babies can be discharged home from the hospital [1,2]. However, the majority of premature babies do not reach the neurological maturity required for independent and safe feeding until the time of their expected date of birth [1,3]. Therefore, even late premature infants usually require a feeding tube to ensure adequate enteral nutrition until they are able to breastfeed or drink from a bottle [4]. Most hospitals have a policy to only discharge premature infants when they no longer need a feeding tube [5]. This often prolongs their hospital stay by days to weeks, at a high cost to the healthcare system [6]. The pressure to learn to drink as quickly as possible in order to be discharged home, and the psychological stress of eventual long hospital stays, may not only harm the child, but also the parents and the family in different ways [7,8,9]. 

It is common practice in neonatology to use the premature infant’s drinking volume alone as a criterion for deciding whether or not a feeding tube is needed [1,3]. A few years ago, the Department of Neonatology at the University Clinic for Pediatrics and Adolescent Medicine in Salzburg also investigated the quality of infants’ drinking behavior. It was found that the drinking quality of numerous premature babies was still quite poor weeks before the expected birth date [8,9,10]. When not only the amount of drinking, i.e., the quantity, but also the quality of the drinking process was considered, the period in which a feeding tube was indicated was significantly prolonged. As it is easier to get a baby to drink from a bottle than from the breast, more parents tended to bottle-feed in order to shorten the hospital stay. Since we commonly also offer feeding tubes for late preterm infants, the rate of breastfeeding clearly increased.

With the knowledge of the sequelae caused by forcing premature infants to drink, an increasing number of newborns have been discharged home with a feeding tube from the University Hospital Salzburg. The primary goal was and is to avoid prolonging the hospital stay, despite the continued need for a feeding tube. Neonates should have the supportive feeding tube available until independent oral feeding is possible with sufficient quality [8,9]. 

As this management showed good results in very small preterm infants, this procedure was extended to late preterm infants. Cardiorespiratory-stable late preterm infants of 34 + 0 weeks’ gestation are admitted with their mothers to the maternity ward and not to a neonatal ward. If a feeding tube is required, they are discharged to home care with a feeding tube. Only if the parents are very unsure or whether additional problems will arise are they transferred to the neonatal ward and discharged from there.

The decision as to when a child can be discharged is made solely based on medical grounds. There is no fixed weight or gestational limit. The children must be cardiorespiratory-stable and must not have any relevant hyperbilirubinemia. In the case of hyperbilirubinemia requiring treatment, phototherapy is also carried out in the postnatal ward. In general, of course, sufficient weight development of the child must be ensured. The most important thing, however, is that the parents are able to deal with their child. If necessary, this also includes handling the feeding tube.

To ensure this, all mothers in the ward receive detailed breastfeeding and lactation advice from specially trained and certified nursing staff. At the same time, parents receive detailed training on how to use the feeding tube in good time before discharge. There are special training documents for parents, and the instruction is standardized. For the period following discharge, necessary equipment such as feeding tubes, fixation plasters, syringes and the like are procured via a pharmacy, and outpatient pediatric nursing (MOKI) is organized for outreach follow-up care. The mobile pediatric nurse visits the family initially on a daily basis and subsequently as required [8,9]. In addition, parents are also offered short-term support via our hospital’s breastfeeding outpatient clinic. The planning of feeding and weaning from the feeding tube is carried out by the mobile pediatric nursing service in close consultation with the neonatology department. Regardless of their drinking behavior, premature babies with a birth weight <2500 g always receive an outpatient neonatal follow-up check approx. 4–6 weeks after discharge, i.e., usually around the calculated date of birth. During this check-up, the children’s growth data are collected, further nutrition is planned with the mothers and a blood sample is taken to check the iron and bone metabolism.

The aim of this paper is to show that it is possible and safe to care for premature babies with a feeding tube in the maternity ward and discharge them to home care. This is illustrated by the length of hospital stay and wellbeing of infants after discharge compared to preterm infants discharged from the maternity ward without feeding tubes.

## 2. Materials and Methods

This study is based on a retrospective analysis of a cohort of late preterm infants born between 1 January 2019 and 31 December 2020. Patients were identified based on their main diagnosis at discharge. All preterm infants with the ICD-10 code P07.32 (other born before term, gestational age 33 to 37 weeks) who were cared for only in the maternity ward and discharged directly to home care were included in the study.

All preterm infants born from 34 + 0 to 36 + 6 weeks’ gestation between 1 January 2019 and 31 December 2020, who were subsequently cared for only in the maternity ward and discharged to home care, were included in the study. Preterm babies who could not be discharged home directly but had to be transferred to another postnatal unit for a prolonged stay were excluded.

For the preterm infants included, it was determined whether they could be discharged from hospital with or without a feeding tube. Based on this distinction, the cohort was divided into two groups. The length of and complications during hospital stay and the thrive until the first outpatient follow-up were compared between the groups. 

The growth data after hospital discharge were obtained either from the outpatient neonatal follow-up or from the outpatient contact at the breastfeeding outpatient clinic.

However, since thriving cannot be determined by a single parameter, demographic data such as gestational age, weight, length and head circumference, as well as clinical data such as hyperbilirubinemia, length of hospital stay, growth development and any re-hospitalizations up to the first outpatient follow-up were statistically compared.

The Ethics Committee of Salzburg approved the study with the number 1046/2023 on 26 April 2023.

Statistics: An unpaired *t*-test was used for numerical values and the chi-squared test for nominal data. A *p*-value < 0.05 was considered statistically significant. Microsoft Office Excel (V2.82 (24020533) for I-Pad) and Primer of Biostatistics (V7 of 2011) were used for statistical analysis.

## 3. Results

**Figure 1:**
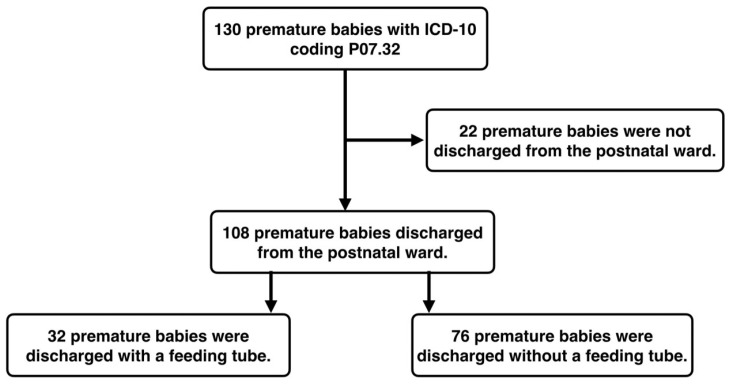
Flowchart of patient recruitment.

Between 1 January 2019 and 31 December 2020, 130 premature babies with the main diagnosis P07.32 were cared for. Of these, 108 premature babies were cared for exclusively at the maternity ward and could also be discharged home with their mothers. Of these, 32 premature babies with a feeding tube and 76 premature babies without a feeding tube were discharged home. The recruitment process of the patients can be seen as a flow diagram in Figure 1.

The mean gestational age at birth was 35.23 weeks (±0.884) for preterm infants with a feeding tube and 35.97 weeks (±0.702) for preterm infants without a feeding tube (*p* = <0.001). The mean birth weight was 2423 g (±375) in preterm infants with a feeding tube and 2588 g (±425) (*p* = 0.084) in preterm infants without a feeding tube. Other demographic data at birth are shown in Table 1.

The mean length of hospital stay was 7.22 days (±3.63) for preterm infants with a feeding tube and 6.82 days (±7.11) (*p* = 0.762) for preterm infants without a feeding tube. The mean discharge weight for preterm infants with a feeding tube was 2365 g (±285) and for preterm infants without a feeding tube, it was 2560 g (±409) (*p* = 0.016). The clinical data during the hospital stay and the demographic data at the time of discharge are shown in Table 1.

At the time of the first outpatient follow-up, the mean weight of preterm infants with a feeding tube was 3466 g (±591), and it was 3784 g (±622) (*p* = 0.064) for preterm infants without a feeding tube. All preterm infants with feeding tubes had their feeding tubes removed by the time of their first outpatient follow-up. None of the preterm infants had relevant feeding difficulties at the time of the first outpatient visit. The demographic data at the time of re-presentation are shown in Table 1.

**Figure 2:**
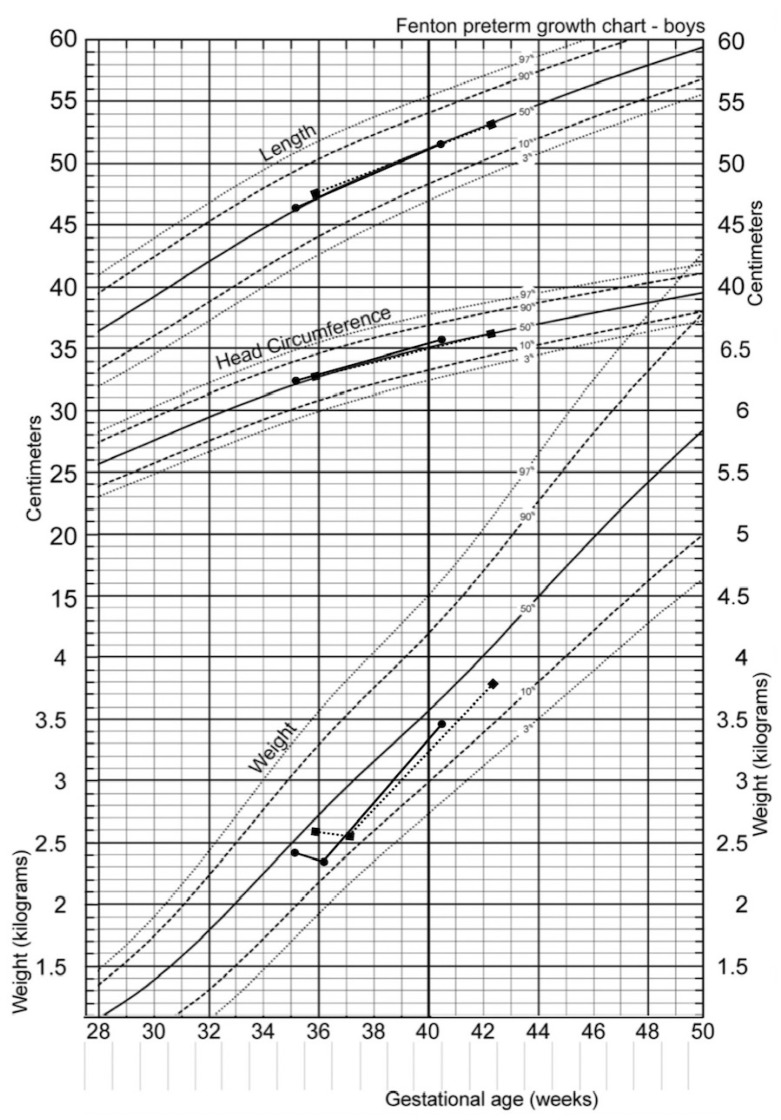
Average growth of preterm infants discharged with a feeding tube (circles with the solid line) and preterm infants discharged without a feeding tube (squares with the dotted line). The mean values were entered into a Fenton growth percentile for boys due to the preponderance of boys in the groups. (11) The body weight was documented at birth, hospital discharge and outpatient follow-up. The head circumference and length were documented at birth and outpatient follow-up.

The respective progression of the weight, head circumference and length of the preterm infants, along the percentile curves with and without a feeding tube at the time of birth, discharge and first outpatient visit are graphically shown in Figure 2. A Fenton curve for male infants was used for this purpose, as the majority of preterm infants are boys [11]. Only the mean values were entered; the statistical comparison of the groups is listed in Table 1.

## 4. Discussion

The neonatal unit in Salzburg has set itself the goal of educating parents to competently take care of their premature infants, therewith they are discharged home as soon as medically possible. Therefore, feeding tubes were left in place in physiologically healthy preterm infants with immature sucking–swallow–breathing coordination until the preterm infants were able to drink well and safely, not only in terms of the quantity, but also in terms of the quality [8,9]. In the period from 1 January 2019 to 31 December 2020, a total of 32 preterm infants with feeding tubes were discharged home directly from the maternity ward in Salzburg. The results we presented demonstrate that the discharge of preterm infants from the maternity ward with a feeding tube is possible and does not lead to an increased risk for preterm infants. 

At an average of 35.2 weeks’ gestation, the preterm babies discharged with a feeding tube were significantly less mature at birth than the preterm babies discharged without a feeding tube at an average of just below 36 weeks’ gestation. The lower the gestational age of the premature babies at birth, the more immature they are. The neurological maturity required for sufficient suck–swallow–breathing coordination (SSBC) is not reached until 32–35 weeks of gestation [10], which means that premature babies are not yet able to drink and therefore require a feeding tube. Full oral feeding is often not achieved until weeks later [3]. As the length of hospitalization and the time between discharge and outpatient visit was the same in both groups, the preterm infants with a feeding tube were also significantly younger than the infants without a feeding tube at the time of discharge and at the time of re-presentation. In her study, Sitter C. concluded that complete oral feeding is only possible from an average gestational age of 37.1 weeks [12]. The preterm infants without a feeding tube were discharged at around 37 weeks’ gestation and were therefore able to develop sufficient SSBC during their stay in hospital. The preterm infants with a feeding tube were almost one week younger at discharge and therefore had a less developed SSBC and had to be discharged with a feeding tube in order not to prolong the hospital stay. 

In relation to the gestational age at birth, the weight in both groups was at the 37th percentile and therefore exactly at the same percentile. However, the weight of the two groups was no longer in the same percentile at the time of the first outpatient visit. The weight of the premature babies with feeding tubes was at the 34th percentile, and the weight of the babies without feeding tubes was at the 30th percentile. Therefore, the infants with additional tube-feeding showed better weight gain despite the fact that the infants had a lower birth weight compared to the infants discharged without tube-feeding. Overall, both groups also showed normal and age-appropriate growth in terms of the head circumference and body length. Basically, the two groups with different initial values in terms of the gestational week and birth weight are compared here. However, it is noticeable that the immature children who were discharged with a feeding tube grew slightly better at the time of the first follow-up examination than the initially more mature children who were discharged without a feeding tube. Of course, this is not proof that discharge with a feeding tube is actually an advantage for children. But what we can say from the available data is that a relatively early discharge with a feeding tube is certainly not a disadvantage for these children and families. 

Collins CT et al., investigated 88 preterm infants with feeding tubes who either remained at the hospital until they were able to drink on their own or were part of an early discharge program with home feeding. There was no significant difference in weight gain between the two groups [5]. In the cohort studied, preterm infants discharged with a feeding tube did not have significantly longer hospital stays than preterm infants discharged without a feeding tube. On average, both groups stayed at the hospital for about 7 days. McLaurin et al., also looked at the average length of hospital stay for 1683 late preterm babies. In their study, Collins CT et al., showed that infants who participated in an early discharge program with home tube-feeding had a hospital stay that was 9.3 days shorter than infants who were discharged with the tube removed [5]. The literature therefore supports our hypothesis that in this patient group, discharge with a feeding tube can shorten the inpatient stay.

All preterm infants observed in this study had their feeding tube removed by the time of the first outpatient visit, and none of the preterm infants had relevant feeding difficulties at that time. The outpatient follow-up was carried out around the calculated date of birth. This means that all children were able to drink their own food without any problems from the calculated date of birth. None of the children had therefore developed a relevant eating disorder. In addition, the preterm infants with feeding tubes had no readmissions (0%), whereas the preterm infants discharged without feeding tubes had a readmission rate of just under 4%. In their study, Darjan Kardum et al. examined the rehospitalization rate of 5408 newborns with a gestational age of ≥36 weeks. With an average rehospitalization rate of 4%, this was identical to our rehospitalization rate for preterm infants without a feeding tube. The preterm infants with a feeding tube had a 0% rehospitalization rate, which was lower than the average of the cited study. Discharge with a feeding tube therefore indicates a lower risk of rehospitalization for preterm infants. 

However, it is important to point out that the department of the Salzburg General Hospital has built up a well-developed infrastructure for the discharge of preterm infants with feeding tubes. The case management forms a well-functioning aftercare program which includes special-trained medical staff at the maternity ward, an educational program for the parents, the Mobile Children’s Nursing Service (MOKI) which sees the families up to several times a week, and an outpatient follow-up clinic. We ensure that preterm infants with feeding tubes are discharged into a well-trained home environment with professional aftercare [8,9]. In terms of the outcomes, the good infrastructure in Salzburg has certainly contributed to the fact that not a single child had to be readmitted to hospital after being discharged with a feeding tube.

An increase in bilirubin is physiological in all newborns. If the level is above the age-related limits, phototherapy must be used to reduce the bilirubin level to prevent serious complications [13]. Late preterm infants have a higher risk of hyperbilirubinemia than term infants [14]. In our study, there was no significant difference in either maximum bilirubin levels or phototherapy rates between preterm infants with and without feeding tubes. At 33% and 38% respectively, the treatment rate in both groups was well below the 54% reported by Sharma et al. [14]. Conjugated bilirubin is generally excreted in the bile and therefore in the stool [13]. Adequate excretion is only possible with adequate food intake. Enteral nutrition is therefore an important factor in preventing hyperbilirubinemia requiring treatment in newborns [15]. The feeding tube ensures adequate enteral nutrition and, by that, allegedly leads to no increased incidence of hyperbilirubinemia requiring treatment in this group.

In our study, there was no significant difference in the gender distribution between the groups with and without feeding tubes. K Herz et al., studied 528 late preterm infants with regard to their neonatal outcome and compared them according to sex. They also concluded that the fetal sex had no effect on neonatal outcomes in the group of preterm infants born at 34–36 + 6 weeks’ gestation. The result is therefore consistent with our finding of no sex difference in the data we collected [16]. 

One limitation of the study lies in the context of the retrospective data collection. One problem is that not all preterm infants had a standardized outpatient follow-up examination. This mainly concerns premature babies with a birth weight of more than 2500 g without a feeding tube. These are usually only followed-up for a few days on an outpatient basis by the lactation consultant, but no scheduled outpatient follow-up appointment is made. Therefore, the data for preterm infants without feeding tubes at the time of outpatient follow-up are not complete. Another limitation to be considered when interpreting the results is that not all premature babies in Salzburg are discharged from the maternity ward to home care at the same time as their mother. Of course, smaller premature babies born before 34 + 0 weeks are sometimes cared for many weeks at the neonatal intensive care unit. And quite a few of the larger preterm babies from 34 + 0 weeks are also transferred from the maternity ward, where they are primarily admitted, to a normal neonatal care ward. This usually happens when mothers are discharged from the maternity ward and the babies still need care. As many premature babies with feeding tubes are discharged from both the intensive care unit and normal care unit, the children presented here certainly do not represent all premature babies discharged with feeding tubes in Salzburg during the period studied.

Another very important point of interest is the long-term follow-up of late preterm babies. It would be very important to know whether preterm infants with the need for a feeding tube show any kind of altered feeding behavior at the time of introducing complementary feeding and beyond. Further studies are needed.

## 5. Conclusions

A lack of recommendations and experience in discharging premature infants with a feeding tube is often the reason why many neonatal units discharge infants only after the feeding tube has been removed, meaning that they often have to stay at the hospital for days or weeks longer [3]. However, this study showed that premature infants with feeding tubes can be discharged at the same time as premature infants who do not require a feeding tube. Therefore, immature drinking behavior does not necessarily prolong the hospital stay. The premature babies continued to benefit from the feeding tube despite being discharged. The weight development was positive and none of the babies had to be readmitted to hospital for failure to thrive. The children concerned were healthy, immature premature babies who all lost their feeding tubes after reaching the necessary maturity. It can therefore be ruled out that the discharge of children with feeding tubes will certainly not provoke long-term eating disorders. The data show that it is possible to discharge preterm infants with a feeding tube from the neonatal unit to home care, and that this is not associated with an increased risk for preterm infants [8,9]. A discharge to a trained and qualified environment can ensure the appropriate thriving of preterm infants without an additional risk.

## Figures and Tables

**Table 1:** Demographic and clinical data of preterm infants included in the study at birth, including hospital discharge and outpatient follow-up. A *p*-value < 0.05 was considered statistically significant (bold).

	Discharged with a Feeding Tube		Discharged without a Feeding Tube		
	n = 32		n = 76		
Birth	Mean	SD	Mean	SD	*p*-Value
Gestational age (weeks)	35.23	0.884	35.97	0.703	**<0.001**
Birth weight (g)	2423	375.1	2589	424.8	**0.048**
Percentile (z-score)	37 (−0.32 z)		37 (−0.32 z)		
Head circumference (cm)	32.6	1.48	32.8	1.56	0.439
Percentile (z-score)	61 (0.28 z)		54 (0.1 z)		
Body length (cm)	46.5	2.45	47.7	2.21	**0.013**
Percentile (z-score)	53 (0.07 z)		59 (0.23 z)		
Female (n)	14 (44%)		35 (46%)		0.994
**Discharge**	Mean	SD	Mean	SD	*p*-value
Gestational age (weeks)	36.26	0.949	36.95	1.227	**0.006**
Length of hospital stay (days)	7.2	3.63	6.8	7.11	0.762
Highest bilirubin value (mg/dl)	13	2.99	12.6	3.53	0.602
Phototherapy performed (n)	12 (38%)		25 (33%)		0.812
Weight at discharge (g)	2365	294.7	2560	409.1	**0.016**
Percentile (z-score)	16 (−0.98 z)		19 (−0.89 z)		
**Outpatient follow-up**	Mean	SD	Mean	SD	*p*-value
Age (days)	38.8	12.05	43.9	15.93	0.215
Gestational age (weeks)	40.43	2.0	42.21	2.554	**0.008**
Weight (g)	3466	591.3	3785	621.8	0.064
Percentile (z-score)	34 (−0.41 z)		30 (−0.52 z)		
Head circumference (cm)	35.8	1.78	36.3	1.45	0.128
Percentile (z-score)	63 (0.34 z)		54 (0.1 z)		
Body length (cm)	51.5	2.00	53.0	2.24	**0.002**
Percentile (z-score)	49 (−0.02 z)		48 (−0.05 z)		
Rehospitalizations	0 (0%)		3 (4%)		0.637

## Data Availability

All data on which the study is based are available in anonymized form via the corresponding author due to (due to ethical restrictions).
